# Late Oligocene–Early Miocene magnetochronology of the mammalian faunas in the Lanzhou Basin–environmental changes in the NE margin of the Tibetan Plateau

**DOI:** 10.1038/srep38023

**Published:** 2016-11-30

**Authors:** Peng Zhang, Hong Ao, Mark J. Dekkers, Yongxiang Li, Zhisheng An

**Affiliations:** 1 State Key Laboratory of Loess and Quaternary Geology, Institute of Earth Environment, Chinese Academy of Sciences, Xi’an 710075, China; 2 Paleomagnetic Laboratory ‘Fort Hoofddijk’, Department of Earth Sciences, Faculty of Geosciences, Utrecht University, Budapestlaan 17, 3584 CD Utrecht, The Netherlands; 3 State Key Laboratory of Continental Dynamics, Department of Geology, Northwest University, Xi’an 710069, China

## Abstract

A large number of terrestrial mammalian fossils were reported in the well-exposed Paleogene and Neogene fluvio-lacustrine strata in Western China. Their accurate ages are crucial to understand the mammalian and environmental evolution associated with the step-wise uplift of the Tibetan Plateau. At present their ages are surprisingly poorly constrained. Here, we present a high-resolution magnetostratigraphic dating of the Late Oligocene–Early Miocene mammal assemblages from a 233-m thick fluvio-lacustrine section in the Lanzhou Basin located at the northeastern margin of the Tibetan Plateau, China. The results suggest that the section spans from the polarity subchron C6Cn.2r to C5En, i.e. ranging from *ca* 23 to 18 Ma. This magnetochronology provides considerably more robust ages for three associated land mammalian faunal assemblages. Updated ages end the debate on existing ambiguous and distinctly different magnetostratigraphic correlations for those Late Oligocene–Early Miocene assemblages. The new ages now enable precise correlation of these faunas to the European Land Mammal and North American Land Mammal Ages. The faunal assemblages further suggest a mixed setting of woodlands and grasslands associated with a humid environment in the Lanzhou Basin during the Late Oligocene–Early Miocene, in contrast to its modern poor vegetation cover and arid environment.

Presently, the Cenozoic continental environmental and mammal evolution of North America, Europe, and Africa have a much higher dating resolution and are better known than that of Asia. From a time-scale viewpoint, the Asian Land Mammal Ages (ALMA) are of distinctly poorer quality than the European Land Mammal Ages (ELMA) and North American Land Mammal Ages (NALMA), not to mention the basic documentation of fossil occurrences along with their biostratigraphic and evolutionary significance^1^,^2^,^3^. Although long continuous terrestrial outcrops with rich mammalian faunas throughout the Cenozoic are relatively common in Asia, only a few long-term records of paleoenvironment and faunal assemblages are actually well-dated^4^,^5^,^6^,^7^,^8^. Other studies report on either general environmental or mammal evolution, however, with rather poorly constrained ages^9^,^10^,^11^,^12^, or just a magnetostratigraphy of terrestrial records without mention of specific paleoenvironmental and paleoclimatic implications^13^,^14^,^15^. Recently, several integrated paleoenvironmental and paleoclimatic studies based on high-resolution ages have been published, which unveil the specifics of environmental systems in the Cenozoic of Asia to an increasing extent^4^,^5^,^16^,^17^,^18^,^19^,^20^. However, a comprehensive understanding of long-term environmental and mammal evolution throughout continental Asia during the early Cenozoic, and their potential linkages with global climate change and regional tectonic processes (e.g., uplift of the Tibetan Plateau) cannot be achieved without more of such well-dated integrated studies.

Many sites rich in mammalian faunas of Oligocene–Miocene age were collected from the fluvio-lacustrine sequences in the Lanzhou Basin located at the northeastern margin of the Tibetan Plateau in Western China (Fig. 1); importantly a basic biochronology has been established^2^,^21^,^22^,^23^. Because these Cenozoic fluvio-lacustrine sediments are devoid of suitable material for radiometric dating, magnetostratigraphy has been used to numerically date these faunas^2^,^17^,^24^. To this end, a high-resolution magnetostratigraphy with a well-established polarity sequence that can be unambiguously correlated to the geomagnetic polarity time scales (GPTS) is required. Only then these faunas can be dated with a resolution of ca. 10^5^ yr within a biogeographic province. In order to precisely date these faunas in the Lanzhou Basin, a first magnetostratigraphic record (Fig. S1) involving the fossiliferous Duitinggou (DTG) section in the southeastern Lanzhou Basin was obtained from the literature^25^. However, this magnetostratigraphy was of comparatively low-resolution with large stratigraphic intervals between sample levels (1–5 m). In retrospect, many polarity chrons were missed when trying to correlate the section to the GPTS in an attempt to formulate a biochronology (Fig. S1). This precluded unequivocal correlation to the GPTS, thus distinctly different magnetostratigraphic correlations and age estimates for the faunas have been suggested^2^,^21^,^24^,^25^ (Fig. S1). In recent years more paleomagnetic records from the Lanzhou Basin (e.g., Xingjiawan^17^ and Fenghuangshan section^26^) and the adjacent Xining Basin^8^,^14^ became available. Thus, the basin’s depositional history and the specific features of its detrital remanent magnetism are better understood. For instance, the recent magnetostratigraphic dating of the Late Miocene Xingjiawan Fauna in the northwest Lanzhou Basin has provided an age model for the upper part of the fossiliferous Xianshuihe Formation^17^. With these new results in mind, here we report on a high-resolution magnetostratigraphic study of the fossiliferous DTG section, aiming to provide precise ages for the associated mammalian faunas and to close the current debate on their ambiguous and unclear ages.

The Lanzhou Basin is a north-northwest trending syncline. It is situated north and northwest of the city of Lanzhou, covering an area of about 300 km^2^ (Fig. 1)^21^. The DTG section (36°13′ N, 103°37′ E), studied here, is located on the eastern limb of the syncline, and has a bedding attitude of strike ~170° and dip ~30° to the west. The section consists of three Formations: the Oligocene–Miocene Xianshuihe Formation is underlain by the Eocene–Early Oligocene Yehucheng Formation and subsequently by the Paleocene–Early Eocene Xiliugou Formation. The lower Formation (i.e. the Xiliugou Formation) consists of red massive sandstone and gravelly sandstone layers, representing a fan-delta system^27^. The overlying Yehucheng Formation is typified by distinctive dark red gypsiferous mudstone, siltstone, and sandstone units, which are considered shallow lacustrine playa sediments deposited in an arid climate^28^. The Xianshuihe Formation at the top mainly consists of red mudstone, intercalated with some sandstone packages (Figs 2 and S2), corresponding to a distal alluvial fan to mudflat environment^27^,^28^,^29^. The Xianshuihe Formation is a rich source of Oligocene–Miocene mammalian fossils and can be further subdivided into three parts: the lower, middle and upper Xianshuihe Formation respectively^22^,^25^,^30^,^31^. Up to now, only a few Eocene mammalian fossils are known from the underlying Yehucheng Formation^21^,^23^, while no mammalian fossils have yet been found in the Xiliugou Formation.

This study focuses on the fossiliferous Middle Xianshuihe Formation, well exposed in the DTG section, which has a stratigraphic thickness of 233 m (0 m stratigraphic level indicates the top of the section). The Miaozuizi (MZZ) Fauna (site GL8801, 36°12′46″ N, 103°36′37″ E) and DTG Fauna (site GL9304, 36°13′4″ N, 103°36′15″ E) occur in the sandstone packages at 201.9–233.4 m and a sandstone layer 29–33.6 m in the DTG section, respectively. The Zhangjiaping (ZJP) Fauna was excavated in the village of Zhangjiaping (~3 km west of the DTG section) (cf. Fig. 1 for the location). The packages of white gravelly sandstone (yielding the MZZ and ZJP Faunas) are important marker beds for within-basin correlation; they can be traced across the entire Lanzhou Basin^2^,^21^,^23^. According to previous studies^2^,^21^,^23^, the fossiliferous layers of the ZJP Fauna can be stratigraphically correlated to the sandstone packages at 119.6–143.3 m in the DTG section. The mammal species of the three faunal assemblages are listed in Table S1.

Here, we collected 390 (sampling interval: 20–50 cm) standard oriented hand specimens from the 233-m thick (outcropping) Middle Xianshuihe Formation in the DTG section for a magnetostratigraphic study. In order to obtain samples that were as fresh as possible, the surface of the outcrop was removed (usually 30–50 cm) to eliminate potential weathering effects and disturbance. Paleomagnetic orientation in the field was done by compass. From each block, two cubic specimens of 2 cm × 2 cm × 2 cm were prepared in the laboratory for stepwise thermal demagnetization. Some left-over materials were used for determination of the magnetic mineralogy.

## Results

Magnetite and hematite dominate the magnetic mineralogy of sediments as suggested by rock magnetic analyses, including temperature-dependent magnetic susceptibility and acquisition curves of the isothermal remanent magnetization (For more detailed information see Figs S3 and S4). For most samples from the DTG section, after progressive removal of a low-temperature component (LTC) at 100–250 °C representing an overprint, a high-temperature characteristic remanent magnetization (ChRM) is isolated during the steps up to 680 °C (Fig. S5). The ChRM decays unidirectionally toward the origin of orthogonal plots. Consistent with a normal polarity LTC overprint, the NRM of reversed polarity samples shows a peak between 150 and 250 °C because the normally-directly LTC is removed during that temperature interval; that of normal polarity samples shows a straightforward decreasing trend from room temperature to 680 °C. In agreement with the aforementioned rock magnetic results (Figs S3 and S4), this indicate the presence of both magnetite and hematite as ChRM carriers (Fig. S5). The same ChRM directions are observed for the 250–580 °C and 600–680 °C parts of the unblocking spectra. Therefore, both hematite and magnetite recorded the same paleomagnetic field when their remanences became locked-in in the sediments. Our observations are fully compatible with acquisition of a detrital remanent magnetization^17^. From the 390 demagnetized levels, 276 (71%) yielded reliable ChRM components (cf. Fig. S6 and Table S2) based on strict selection criteria: (1) at least four (but typically 6–15) consecutive demagnetization steps starting from at least 250 °C (with upper temperatures of ≥450 °C), (2) a maximum angular deviation (MAD) less than 10° for a line fit. A total of 81 samples did not yield reliable ChRM directions because they show unstable demagnetization trajectories at higher temperatures, or maximum angular deviation (MAD) values ≥10° and 33 samples are rejected for the declinations trending roughly north (or south) but with upward (or downward) inclinations inconsistent with the expected geomagnetic field. These excluded samples are associated with both the coarser grained lithologies (siltstone, and sandstone) and fine lithologies (mudstone) (cf. Fig. 2).

Virtual geomagnetic pole (VGP) latitudes were calculated from the 276 ChRM directions. Seven samples that lay >30° from the means of normal and reversed VGP latitudes (open triangles in Fig. 2) were discarded as well, as they may have recorded a transitional geomagnetic field^32^. Finally, the VGP latitudes of the remaining 269 samples are used to establish the polarity sequence of the DTG section (Fig. 2). It allows us to recognize 10 pairs of normal and reversed polarity zones. The 151 normal ChRM directions yield an overall mean of declination D = 9.6° and inclination I = 22.4° (k = 14.1, a_95_ = 3.2°; k is the precision parameter and a_95_ is the radius of 95% confidence cone around the mean direction) before tilt adjustment and D = 1.0 and I = 31.2 (k = 14.2, a_95_ = 3.2°) after tilt adjustment. The 118 reversed ChRM directions yield an overall mean of D = 202.6° and I  = −24.3° (k = 13.6, a_95_ = 3.6°) before tilt adjustment and D = 193.2° and I = −37.7° (k = 14.2, a_95_ = 3.6°) after tilt adjustment. The reversals test is negative at the 95% confidence level^33^ possibly due to variable overlap of a normal polarity overprint with the ChRM directions (cf. Figs S5 and S6).

## Discussion

Although the mammalian faunas excavated in the Lanzhou Basin do not indicate a precise age, they provide an approximate chronology as a starting point for our high-resolution magnetostratigraphy. Consistent with palynological^12^ and lithostratigraphic^27^,^29^ data of the Xianshuihe Formation, the mammalian taxa indicate a Late Oligocene to Early Miocene for the MZZ Fauna, Early Miocene for the ZJP Fauna, and Early to Middle Miocene for the DTG Fauna^21^,^22^,^23^,^31^. The stratigraphical and evolutional significance of these faunas have been amply addressed in previous studies^2^,^21^,^22^,^23^,^31^, and will not be re-iterated here. Combining the Late Miocene magnetostratigraphy of the upper Xianshuihe Formation^17^ with the Early to Middle Miocene age of the DTG Fauna, we can readily correlate the established high-resolution magnetic polarity sequence for the DTG section to the GPTS^1^,^3^ (Fig. 2). The distinctive pattern of two long normal intervals (N1 and N2) separated by a shorter reversed interval (R1) in the uppermost section provides a unique correlation to polarity chrons C5En and C6n. The underlying two normal intervals (N3 and N4) separated by reversed interval R3 can be readily correlated to polarity chrons C6An.1n and C6An.2n. Underneath, the four short normal intervals (N5–N8) separated by three short reversed intervals (R5–R7) correlate to polarity chrons C6AAr.1n, C6AAr.2n, C6Bn.1n and C6Bn.2n. The lowermost two normal intervals (N9 and N10) separated by reversed interval R9 correlate to polarity chrons C6Cn.1n and C6Cn.2n. The reversed interval R10 at the bottom of the section possibly correlates to C6Cn.2r. Therefore, our sampled DTG section spans from the polarity subchron C6Cn.2r to C5En, ranging from *ca* 23 to 18 Ma in age. The Oligocene–Miocene boundary at 23.03 Ma^1^,^3^ is located in a sand layer at the bottom of the middle Xianshuihe Formation at 190 m (Fig. 2). We note that one expected rather short normal polarity subchron (C6AAn) in the GPTS is missing in the DTG section, which might be due to a minor hiatus in the sedimentary record. Sandstone layers occur frequently in this interval (Fig. 2), which are generally associated with sedimentary erosion with minor gaps as a result^34^. This absence of short polarity chrons is rather common in the Cenozoic sedimentary records in western China^8^,^14^,^34^. In addition, variable overprint in the ChRM direction may be a potential cause of a negative reversals test for the Cenozoic Asian terrestrial sediments, however, this problem can be overcome by consistency of the retrieved polarity pattern with the expected pattern of GPTS^4^,^8^ (Fig. 2).

**Figure 1:**
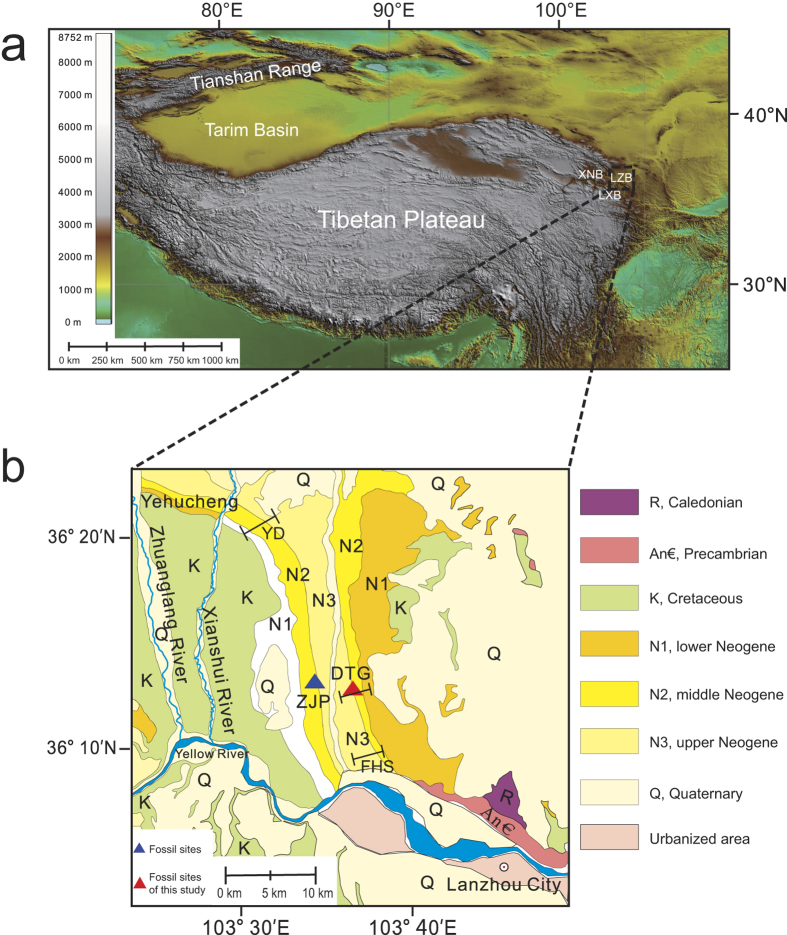
Schematic location and geologic maps showing the Tibetan Plateau and Lanzhou Basin. LXB, Linxia Basin; LZB, Lanzhou Basin; XNB, Xining Basin, YD, Yongdeng section; ZJP, Zhangjiaping Fauna; DTG, Duitinggou section; FHS, Fenghuangshan section. Maps (**a**) and (**b**) were created with QGIS version 2.0.1 (Open Source Geospatial Foundation Project, http://www.qgis.org/en/site/).

The relationship between the stratigraphic thickness and the magnetostratigraphic ages (Fig. 3) shows an almost linear trend in sediment accumulation rates through time without abrupt shifts, testifying to the robustness of the proposed correlation. The Middle Xianshuihe Formation has a relatively low sedimentation rate of 40 m/Myr in the DTG section (Fig. 3). This low sedimentation rate is consistent with that of the nearby Xining Basin (*ca* 30 m/Myr for the Xiejia^14^ and Tashan^8^ sections) and Linxia Basin (*ca* 14 m/Myr for the Maogou section^35^) during the Late Oligocene–Early Miocene (cf. Fig. 1 for their locations).

**Figure 2:**
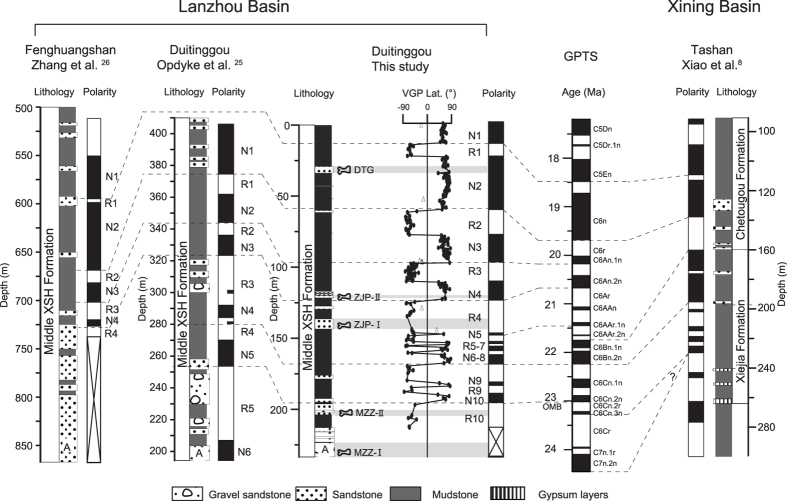
Lithostratigraphy and magnetostratigraphy of the DTG (this study and Opdyke *et al*.^25^) and Fenghuangshan sections^26^ in the Lanzhou Basin and of the Tashan section in the adjacent Xining Basin^8^, and their correlations to the geomagnetic polarity timescale (GPTS)^1^,^3^. The correlations of Fenghuangshan and Tashan magnetostratigraphic records with GPTS are the same as their published studies^8^,^26^, but the previous DTG magnetostratigraphy^25^ (c.f. Fig. S1 for the previous different magnetostratigraphic correlations) is re-interpreted here based on our improved polarity chron structure and regional stratigraphic comparisons. The white gravel sandstone (layer A) at the bottom of the Middle Xianshuihe Formation at both the Duitinggou and Fenghuangshan sections is a sedimentary marker layer with variable thickness across the Lanzhou Basin, which contains MZZ-I Fauna. Open triangles represent discarded samples that lay >30° from the means of normal and reversed VGP latitudes. OMB, Oligocene–Miocene boundary.

**Figure 3:**
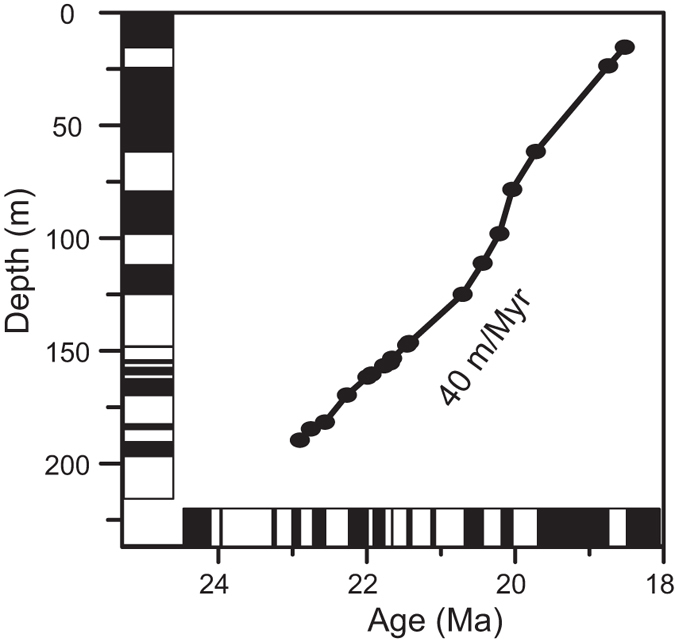
The relationship between the stratigraphic level and the magnetostratigraphic ages in the DTG section.

Our magnetostratigraphic correlation to the GPTS is aided by independent and previously obtained age constraints. In comparison to the initial magnetostratigraphic record from the DTG section^2^,^24^,^25^ and a recent record from the Fenghuangshan section^26^ (6 km to the south of the DTG section), our new detailed magnetostratigraphy considerably improves the polarity chron structure of the Middle Xianshuihe Formation in the Lanzhou Basin, although the three records have roughly similar magnetic polarity sequences (Fig. 2). The upper four normal intervals (N1–N4) separated by three reversed intervals (R1–R3) in the present DTG section and the Fenghuangshan section^26^ are consistently correlated to correlated to C5En–C6An.2n. Possibly because of frequently thick sandstone deposition in the Fenghuangshan section, Zhang *et al*.^26^ failed to establish a reliable magnetostratigraphy for the lower part of the Middle Xianshuihe Formation. The upper three normal intervals (N1–N3) suggested by the study of Opdyke *et al*.^25^ are consistent with the three normal intervals N2–N4 in our study, which should correlate to polarity chrons C6n–C6An.2n rather than C5ADn–C5Bn.2n^24^. The underlying two normal polarity chrons N4 and N5 suggested by Opdyke *et al*.^25^ possibly coincide with the normal intervals N5–N8 and N9–N10 in our record, which should correlate to polarity chrons C6AAr.1n–C6Bn.2n and C6Cn.1n–C6Cn.2n, respectively. This reinterpretation is contrasting with the previously debated assignments of N4 to C6AAn or C5Cn and of N5 to C6AAr.1n or C5Dn^2^,^24^,^25^ (Fig. S1). In addition, the presently established Late Oligocene–Early Miocene magnetochronology from the DTG section in Lanzhou Basin is consistent with that from partly coeval sections in the adjacent Xining Basin^8^, which have a similar polarity structure (Fig. 2). These detailed regional comparisons indicate that high-resolution magnetostratigraphic records, such as the present DTG record, are mandatory to establish unambiguous correlations to the GPTS.

**Figure 4:**
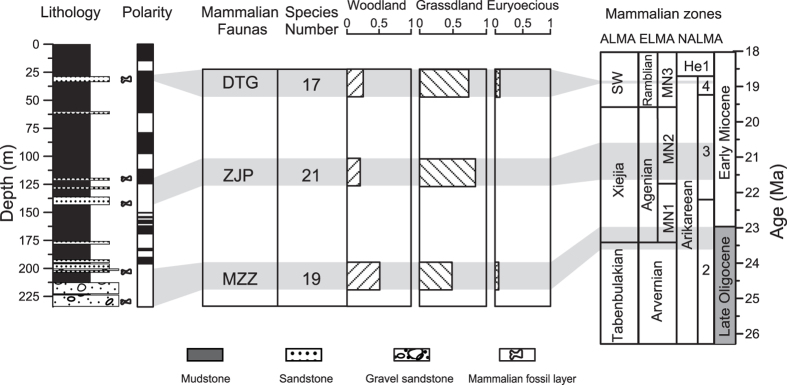
Variability of the total number of different mammalian species and percentages of grassland, woodland and euryoecious species for the dated mammalian faunas in the Lanzhou Basin, and its correlation to the Land mammalian ages of Asia (ALMA), Europe (ELMA) and North America (NALMA)^1^,^3^. SW, Shanwangian; He, Hemingfordian.

The high-resolution unambiguous magnetochronology for the Middle Xianshuihe Formation obtained in our study provides new and robust ages for these mammalian faunas and closes the debate on the ages of mammal faunas associated with the DTG section^2^,^24^,^25^. The numerical ages for the faunas can be obtained through linear interpolation/extrapolation using the ages of appropriate geomagnetic reversal boundaries as age tie points. With this new age frame, these local Lanzhou Basin faunas can be firmly correlated to the established ALMA, ELMA and NALMA^1^,^3^ (Fig. 4). The DTG Fauna is directly excavated from the sandstone package within the uppermost part of the polarity chron C6n, which has an estimated age of 18.9 Ma through linear interpolation of the section’s sedimentation rate in the C6n polarity chron. It is correlated to the Shanwangian stage of ALMA, the MN3 zone of ELMA, and the Ar4 stage of NALMA. Because of imprecise dating in earlier studies, the DTG Fauna was previously inaccurately correlated to the European zone MN4^23^,^31^. The ZJP Fauna contains two fossiliferous layers that are stratigraphically correlated to two sandstone packages at *ca* 119.6–121.3 m and 136.3–143.3 m in the DTG section. The ZJP-II Fauna in the younger package corresponds to the C6An.2n normal polarity chron and yields an age of ~20.6 Ma, while the missing of the C6AAn in zone R4 complicates the establishment of a precise age for the ZJP-I Fauna. However, based on the correlation of N1–N4 and N5–N10, the ZJP-I Fauna should be located between C6An.2n and C6AAr.1n, with an age estimate of *ca* 21.1–21.3 Ma. Thus, the whole ZJP Fauna can be correlated to the middle Xiejia stage of ALMA, the MN2 zone of ELMA, and the latest Ar3 zone of NALMA, respectively. The MZZ Fauna occurs in two conglomeratic sandstone layers at *ca* 201.5–203.6 m and 223.4–233.4 m. The younger MZZ-II Fauna is situated in the upper part of the R10 reversed magnetozone. Linear extrapolation of sedimentation rates of the C6Cn.2n chron provides an age of ca 23.2 Ma for that fauna. The present study did not yield any reliable magnetostratigraphic data from the white gravel sandstone layer at the bottom that contains MZZ-I Fauna. Linear extrapolation of normal polarity chron N10 and the generally rapid deposition of gravel sandstones indicate that the older MZZ-I Fauna might be tied to an age within the reversed polarity chron C6Cr. So, the MZZ Fauna can be roughly correlated to the late Tabenbulakian to earliest Xiejia stage of ALMA, the late Arvernian stage to MN1 zone of ELMA, and the late Ar2 zone of NALMA. Therefore, our more precise magnetostratigraphic dating of these mammalian faunas leads to updated correlations of these faunas (i.e. the DTG, NPP, and MZZ Faunas) to the global Land Mammalian Ages. It provides a chronological base for further study of the mammalian evolution and environmental changes in East Asia during the Oligocene–Early Miocene, and represents a further step toward the establishment of a complete and robust chronology for the Cenozoic ALMA from the viewpoint of the International Land Mammal Stratigraphy commission.

The taxonomic composition and faunal properties of the mammals are sensitive to environmental conditions^17^,^36^,^37^. So, a broad environmental setting during Oligocene–Early Miocene times can in principle be established by investigating these mammal fossils in the Lanzhou Basin. Both typical grassland (e.g., *Tataromys* and *Bounomys* of Ctenodactylidae) and woodland species (e.g., *Schizotherium* (extinct relatives of the horse) and Indricotheriinae (extinct relatives of Rhinocerotidae)) are identified in the mammalian faunas (Table S1). We calculated the mammalian occurrence of the woodland and grassland groups as percentages (Fig. 4). The ZJP (ca 21.7–20.6 Ma), and DTG (18.9 Ma) Faunas appear to be dominated by grassland species, with higher grassland percentage than woodland percentage. The MZZ Fauna (ca 24–23.2 Ma) has equal percentages of woodland and grassland species. As a whole, the abundant mammals, especially some large mammals such as *Paraceratherium* in the MZZ Faunas, together with widespread lacustrine sediments, imply that the Lanzhou Basin was probably warmer and more humid with much denser vegetation during the Late Oligocene–Early Miocene than today to be able to support such grassland and woodland mammals. This environmental inference from mammals is consistent with the Late Oligocene–Early Miocene wet environment suggested by pollen records^12^ and megafossil plants^38^, in contrast to its modern arid environment that is not suitable for large mammals and dense vegetation.

## Methods

About 300 mg powdered samples were used for measuring χ–T curves with a MFK1 FA magnetic susceptibility meter equipped with a CS-3 high-temperature furnace (AGICO, Brno, Czech Republic). Measurements were done in an argon atmosphere from room temperature up to 700 °C and back to room temperature (heating and cooling rate of ~6.5 °C/min). The magnetic field during measurement was 300 A/m (peak-to-peak). The susceptibility of each sample was corrected for the background χ (furnace tube correction) using the CUREVAL 8.0 program (AGICO, Brno, Czech Republic). Four non-magnetic cubic boxes (with an edge of 2 cm) full of powdered sample material were used for the measurement of IRM acquisition curves with an AGICO JR-6A dual speed spinner magnetometer in a magnetically shielded room (residual field <150 nT). IRMs were imparted with an impulse magnetizer (ASC, model IM-10-30). IRM acquisition curves consist of 34 field steps with a maximum field of 2.7 T.

Stepwise thermal demagnetization of the natural remanent magnetization (NRM) was performed using a TD-48 thermal demagnetizer, with increments of 10–50 °C to 680 °C (18 demagnetization steps). After each demagnetization step, the remaining NRM was measured with a horizontal pass-through 3-axis 2-G cryogenic superconducting magnetometer (model 755-R) housed in the same magnetically shielded space mentioned above. The NRM intensity of the samples is usually of the order of 10^−3^−10^−2^ A/m, while the background value is less than 10^−6^ A/m. Samples were fixed on the tray of the horizontal pass-through magnetometer in groups of eight, and we did not rotate or invert the samples during the measurement procedure in the magnetometer. Only individual measurements with drift values of <10^−6^ A/m were used for paleomagnetic analyses; if drift appeared to be higher, samples were re-measured. Demagnetization results were evaluated by orthogonal diagrams^39^; the principal component direction for each sample was computed using a least-squares linear fitting technique^40^. The principal component analysis (PCA) was done using the PaleoMag software developed by Jones^41^; the least-squares linear fits included the origin. All the rock magnetic and paleomagnetic measurements were carried out at the Institute of Earth Environment, Chinese Academy of Sciences, Xi’an.

## Additional Information

**How to cite this article**: Zhang, P. *et al*. Late Oligocene–Early Miocene magnetochronology of the mammalian faunas in the Lanzhou Basin–environmental changes in the NE margin of the Tibetan Plateau. *Sci. Rep.* **6**, 38023; doi: 10.1038/srep38023 (2016).

**Publisher's note:** Springer Nature remains neutral with regard to jurisdictional claims in published maps and institutional affiliations.

## Supplementary Material

Supplementary Information
