# Supplementary material for: Late Oligocene–Early Miocene magnetochronology of the mammalian faunas in the Lanzhou Basin–environmental changes in the NE margin of the Tibetan Plateau
Source: Sci Rep. 2016 Nov 30;6:38023. doi: 10.1038/srep38023 (PMC5128816; doi:10.1038/srep38023)
Supplement: Supplementary Information [file srep38023-s1.doc]

**Supplementary Material**

**Late Oligocene**–**Early Miocene magnetochronology of the mammalian faunas in the Lanzhou Basin—environmental changes in the NE margin of the Tibetan Plateau**

Peng Zhang^1^, Hong Ao^1*^, Mark J. Dekkers ^2^, Yongxiang Li^3^, Zhisheng An^1^

^1^ State Key Laboratory of Loess and Quaternary Geology, Institute of Earth Environment, Chinese Academy of Sciences, Xi’an 710075, China

^2^ Paleomagnetic Laboratory ‘Fort Hoofddijk’, Department of Earth Sciences, Faculty of Geosciences, Utrecht University, Budapestlaan 17, 3584 CD Utrecht, The Netherlands

^3^State Key Laboratory of Continental Dynamics, Department of Geology, Northwest University, Xi’an 710069, China

*Corresponding author, Hong Ao: [aohong@ieecas.cn](mailto:aohong@ieecas.cn)

**Supplementary Figures**

**
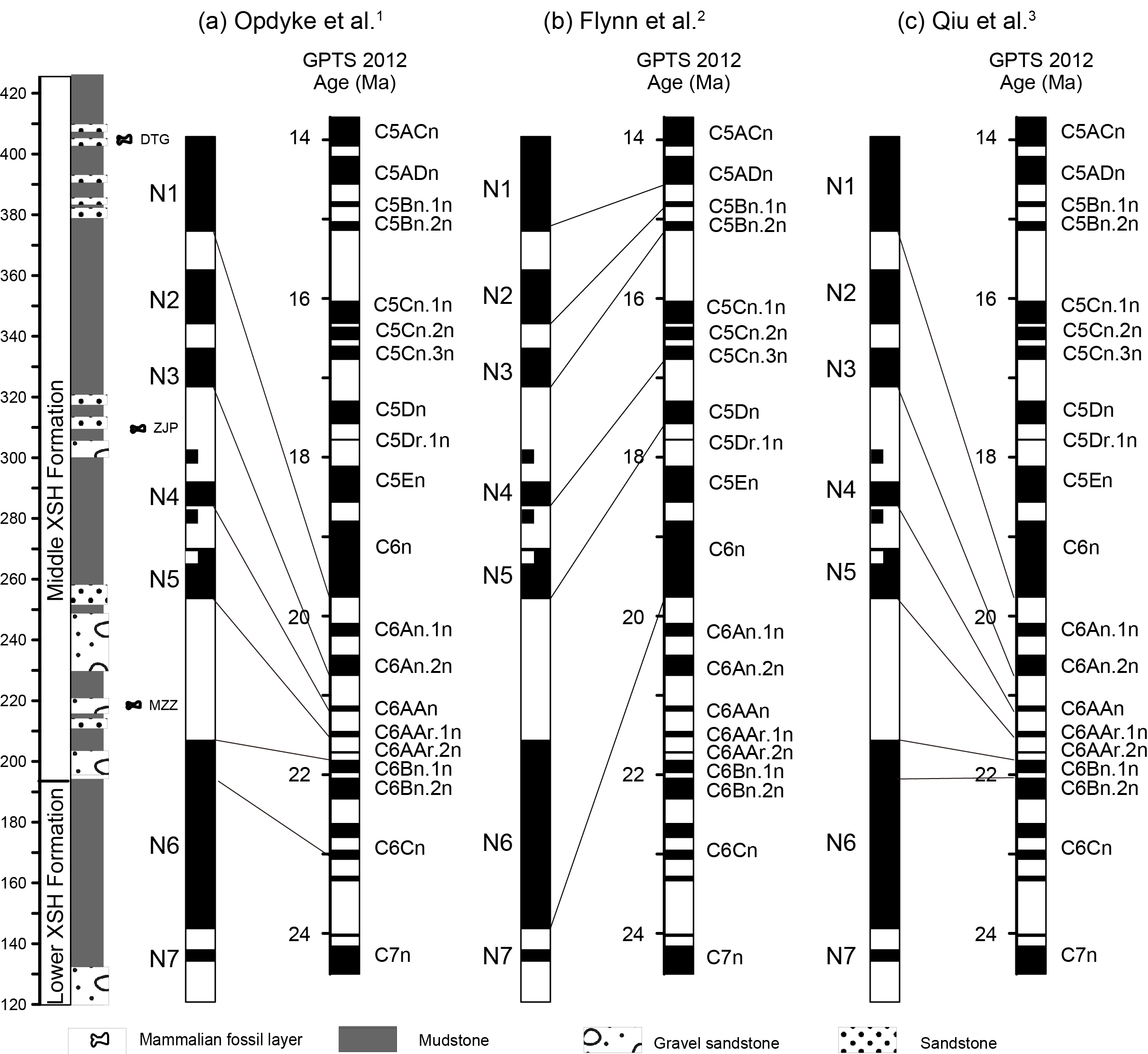
**

Supplementary Figure S1. Magnetostratigraphic correlations of the Duitinggou section^1-3^ to the GPTS^4, 5^. The same magnetostratigraphic record was correlated in different ways to the GPTS due to the comparatively low resolution of the magnetostratigraphy. Therefore, it is well possible that short polarity chrons are missed with an inherently ambiguous interpretation as result.


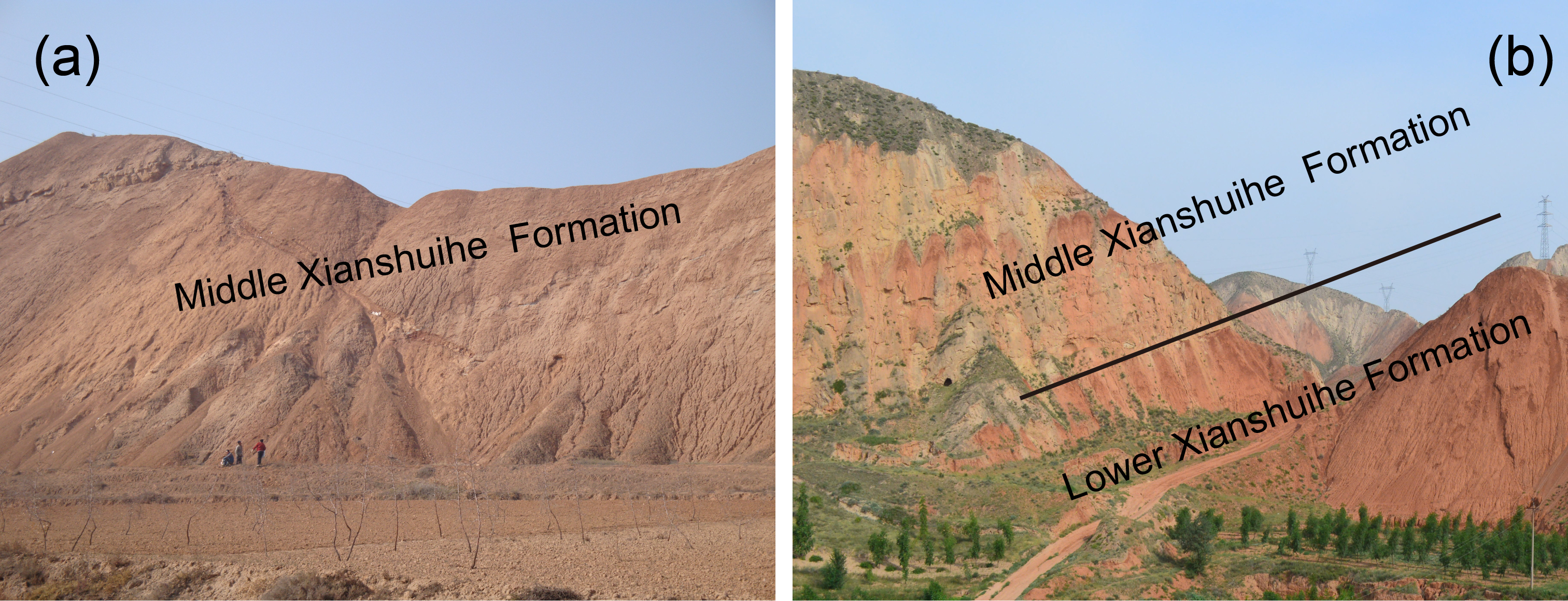


Figure S2. Photographs of the Duitinggou (DTG) section. (a) upper part of the Middle Xianshuihe Formation, and (b) boundary between the Middle and Lower Xianshuihe Formation


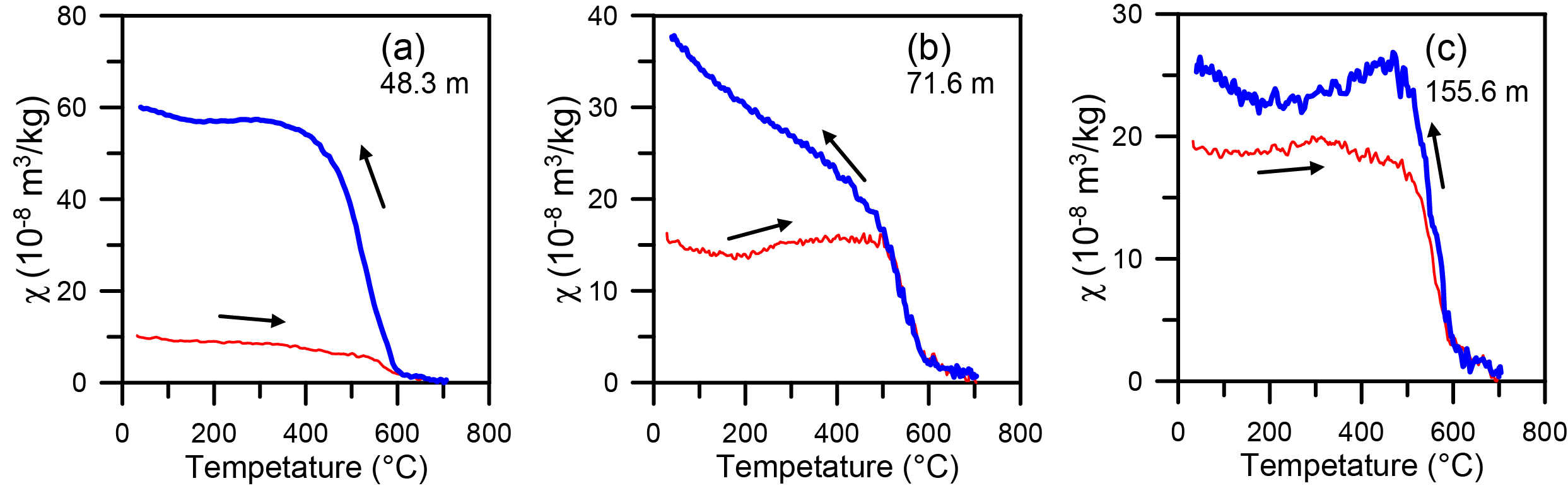


Figure S3. Temperature dependence of magnetic susceptibility (χ–T) of typical samples from the DTG section. Thin red (thick blue) lines represent heating (cooling) curves. The χ–T curves are characterized by a major drop in magnetic susceptibility at 500–600°C, indicating the ubiquitous presence of magnetite in our samples. χ of hematite is about two orders of magnitude lower than that of magnetite. Hematite’s presence is usually masked magnetically by the much stronger contribution of magnetite, thus its expression on χ–T curves is generally subdued when both magnetite and hematite are present. However, the χ–T curves still display a decreasing χ between 580 and 680 °C, which suggests the presence of abundant hematite in these samples, consistent with the red color of the mudstone beds. After heating to 700 °C, the samples show an increased χ during cooling, which is due to neoformation of magnetite during heating^6, 7^.


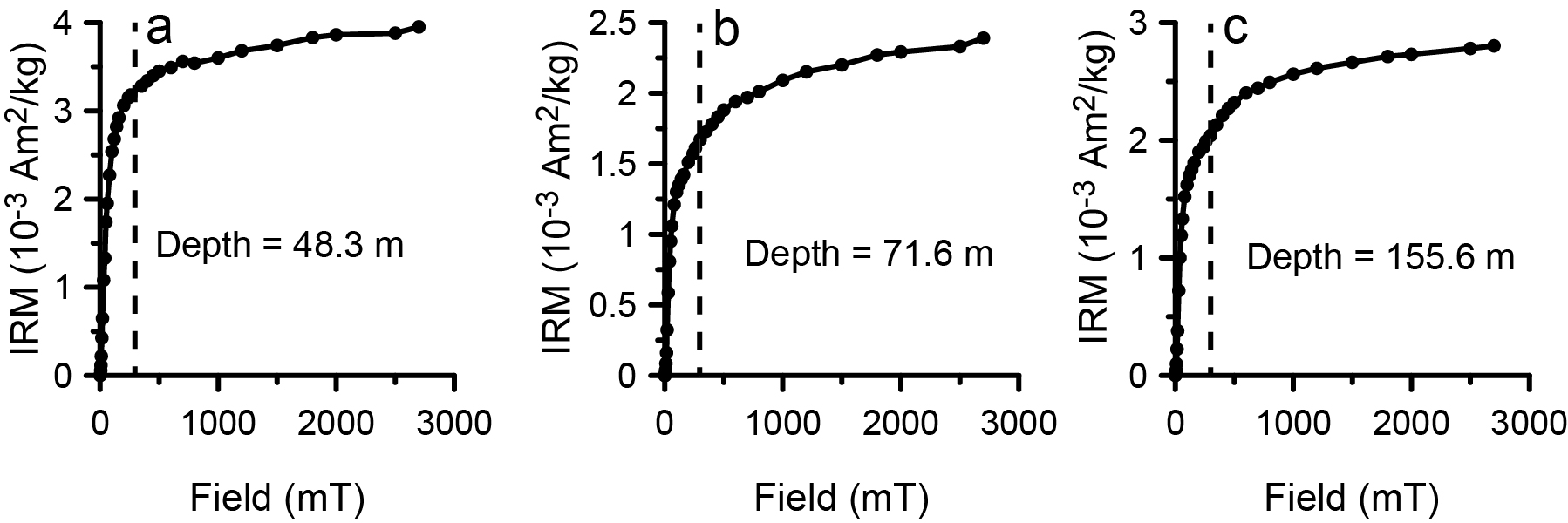


Figure S4. Isothermal remanent magnetization (IRM) acquisition curves of typical samples from the DTG section. The dashed vertical lines at 300 mT are shown to aid distinction between low- and high-coercivity portions of the IRM acquisition curves. All IRM acquisition curves undergo a major increase below 300 mT and the IRM remains unsaturated up to 2.7 T, suggesting the presence of both low-coercivity magnetite and high-coercivity hematite.


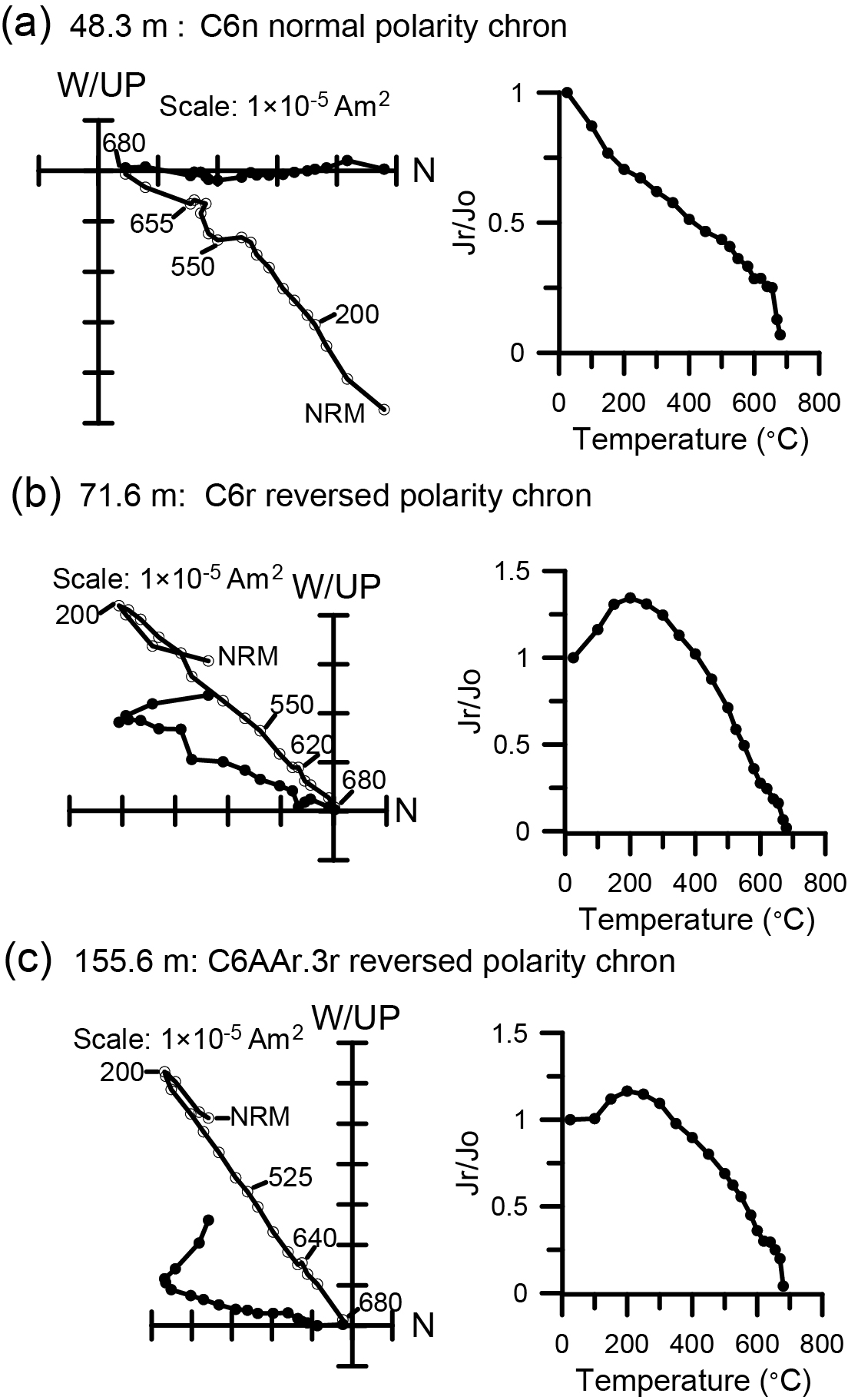


Figure S5. The direction and intensity evolution of the NRM during stepwise thermal demagnetization for selected samples from the DTG section. Solid (open) circles represent the horizontal (vertical) planes. The numbers refer to the demagnetization temperatures in °C and NRM is the starting natural remanent magnetization.


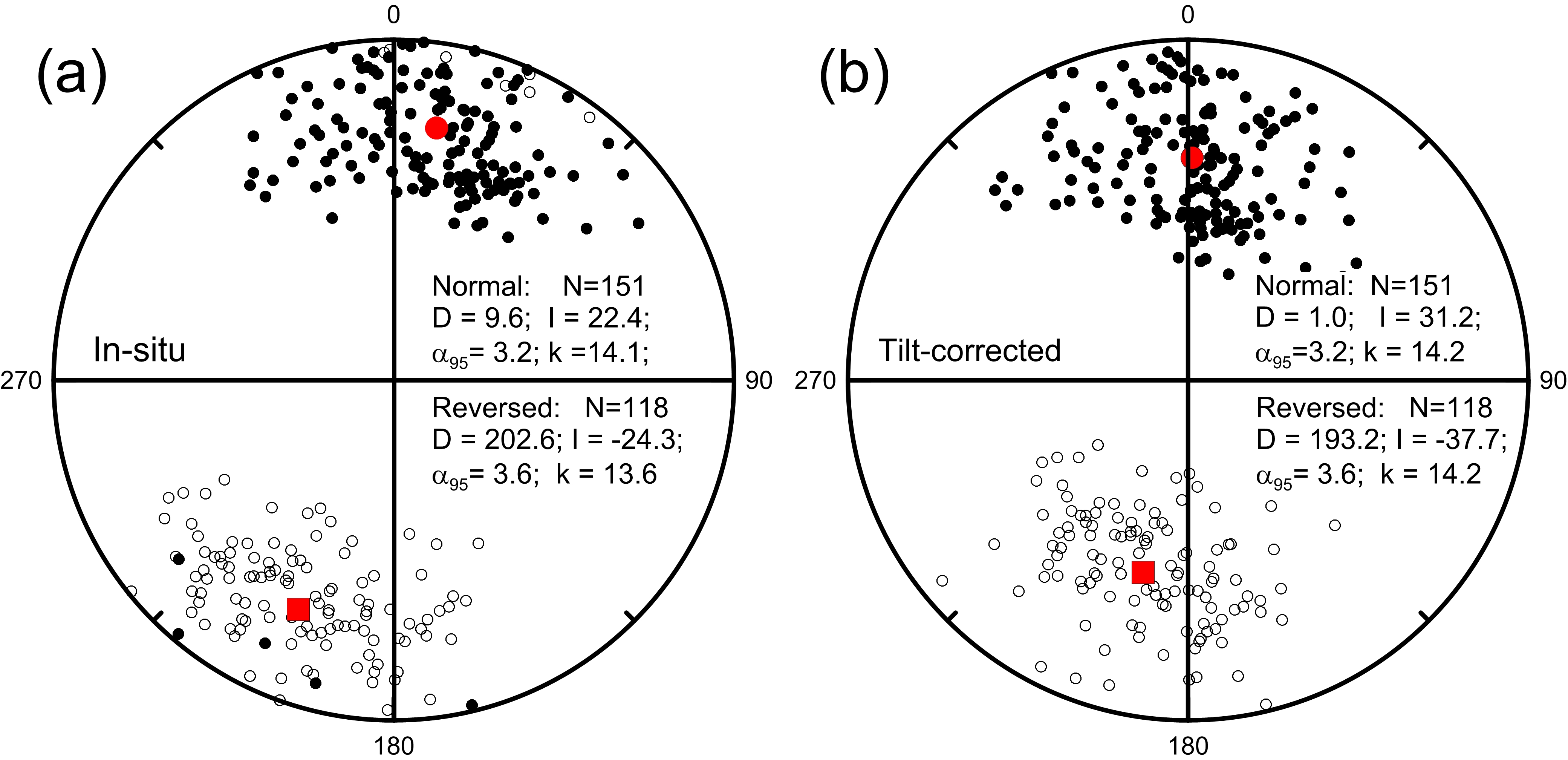


Figure S6. (a) Equal-area projections of 269 ChRM directions before (in-situ) and after (tilt-corrected) tilt correction. Solid/open symbols represent downward/upward inclinations; N, number of data points; D, declination; I, inclination; α_95_, radius of 95% confidence cone around the mean direction; k, precision parameter; red circles (squares), means of normal (reversed) directions. The angular difference between the mean directions of the normal and reversed polarities is 11.9°, slightly larger than the radius of the critical angle (10.1°) at 95% confidence level. The data thus do not pass the reversals test^8^. This may be due to a slight bias of the mean reversed direction towards the west as is the case for the data in the Xining Basin^9^. This small bias may result from incomplete separation of a normal overprint in some of the ChRM directions^9-11^. In any case, this does not compromise the reliability of the polarity determination in the DTG section. Consistent with previously reported paleomagnetic results of Paleogene and Neogene red beds in Asia^12, 13^, most ChRM directions of the DTG section tend to have shallow inclinations (Table S2), which is interpreted to be due to the syn- to post-depositional flattening. This inclination shallowing is a common feature of the Cenozoic red beds in Asia, and is associated with a reliable ChRM^14, 15^.

**Supplementary Tables**

Supplementary Table S1. List of Duitinggou (DTG) Fauna, Zhangjiaping (ZJP) Fauna, and Miaozuizi (MZZ) Fauna in the Lanzhou Basin^3, 16–18^.

| Taxa | MZZ–I  Fauna | MZZ–II  Fauna | ZJP–I Fauna | ZJP–II Fauna | DTG Fauna |
| --- | --- | --- | --- | --- | --- |
| Erinaceidae indet. |  |  |  |  | + |
| *Amphechinus* sp. |  |  |  |  |  |
| *Amphechinus* cf. *A. rectus* |  |  |  |  |  |
| *Amphechinus* cf. *A. minimus* |  |  |  |  |  |
| *Metexallerix* sp. |  |  |  |  | + |
| Soricidae indet. |  |  |  |  | + |
| Talpidae indet. |  |  |  |  | + |
| *Desmatolagus pusillus* (Teilhard, 1926) |  |  |  |  |  |
| *Desmatolagus* cf. *D. gobiensis* |  |  |  |  |  |
| Desmatolagus sp. indet. |  |  |  |  | + |
| Ochotonidae gen. et sp. indet. |  |  |  |  |  |
| Ochotonoides (Teilhard, 1928) |  | + |  |  |  |
| *Alloptox minor* (Li, 1965) |  |  |  |  | + |
| *Ordolagus* sp. |  |  |  |  |  |
| *Ordolagus teilhardi* (Burke, 1941) |  |  |  |  |  |
| *Sinolagomys* sp. | + |  |  |  | + |
| *Sinolagomys kansuensis* (Bohlin, 1937) |  |  |  | + |  |
| *Sinolagomys pachygnathus* (Li et Qiu, 1980) | + |  |  |  |  |
| *Sinolagomys* cf. *S. major* |  |  |  |  |  |
| Aplodontidae |  |  |  |  |  |
| *Tsaganomys* sp. |  | + |  |  |  |
| *Tsaganomys altaicus* (Matthew et Granger, 1923) |  |  |  |  |  |
| Tataromyindae indet. |  |  |  |  |  |
| *Tataromys* sp. | + |  |  | + |  |
| *Tataromys plicidens* (Matthew et Granger, 1923) |  |  | + |  |  |
| *Tataromys sigmodon* (Matthew et Granger, 1923) |  |  |  |  |  |
| *Tataromys minor* (Huang, 1985) |  |  |  |  |  |
| *Tachyoryctoides* sp. | + | + |  |  |  |
| *Tachyoryctoides kokonorensis* (Li et Qiu, 1980) |  |  |  | + |  |
| *Eucricetodon* sp. |  |  |  | + |  |
| *Eucricetodon asiaticus* (Matthew et Granger, 1923) |  |  |  |  |  |
| *Heterosminthus* sp. |  |  |  |  | + |
| *Heterosminthus lanzhouensis* (Wang et Qiu, 2000) |  |  |  |  |  |
| *Parasminthus* sp. |  |  |  | + | + |
| *Parasminthus* spp. |  |  |  |  |  |
| cf. *Parasminthus* | + |  |  |  |  |
| cf. *Parasminthus xiningensis* |  |  |  | + |  |
| *Parasminthus asiae-centralis* ( Bohlin, 1946) |  |  |  |  |  |
| *Parasminthus tangingoli* ( Bohlin, 1946) |  |  |  |  |  |
| *Parasminthus parvulus* ( Bohlin, 1946) |  |  |  |  |  |
| *Sinosminthus* sp. |  |  |  |  |  |
| cf. *Plesiosminthus xiningensis* | + |  |  |  |  |
| *Protalactaga grabaui* (Schaub, 1934) |  |  |  |  | + |
| cf. *Protalactaga grabaui* |  |  |  | + |  |
| *Democricetodon* sp. |  |  |  | + | + |
| *Yindirtemys gobiensis* (Kowalski, 1974) |  |  |  | + |  |
|  |  |  |  |  |  |
| *Yindirtemys* *xiningensis* (Wang, 1997) | + |  |  | + |  |
| *Yindirtemys ambiguous* (Wang, 1997) |  |  |  |  |  |
| *Yindirtemys granger* (Bohlin, 1946) |  |  |  |  |  |
| aff. *Bellatona forsythmajori* |  |  |  | + |  |
| cf. *Bellatona forsythmajori* |  |  |  |  | + |
| *Bounomys* sp. |  |  |  | + |  |
| *Bounomys bohlini* (Huang, 1985) |  |  |  |  |  |
| *Bounomys ulantatalensis* (Huang, 1985) |  |  |  |  |  |
| cf. *Ansomys* sp. | + |  |  | + |  |
| *Monosaulax* sp. |  |  |  |  |  |
| aff. *Mioechinus gobiensis* |  |  |  | + |  |
| *Sinotamias primitivus* (Qiu, 1991) |  |  |  |  | + |
| *Atlantoxerus orientalis* (Qiu, 1996) |  |  |  |  | + |
| *Prodystylomys* sp. |  |  |  |  | + |
| *Anomoemys lohiculus* (Matthew et Granger, 1923) |  |  |  |  |  |
| *Litodonomys huangheensis* (Wang et Qiu, 2000) |  |  |  |  |  |
| *Karakoromys* sp. |  |  |  |  |  |
| *Steneofiber* sp. |  |  |  |  |  |
| *Allacerops* cf. *A. turgaica* |  |  |  |  |  |
| *Sayimys* sp. |  | + |  |  |  |
| *Didymoconus berkeyi* (Matthew et Granger, 1924) |  |  |  |  |  |
| *Plesictis* sp. |  |  | + |  |  |
| *Gobicricetodon* sp. |  |  |  | + |  |
| *Ictiocyon* sp. |  |  |  | + |  |
| Amphicyonidae gen. et sp. indet. |  |  |  |  | + |
| *Hyaenodon* sp. | + | + | + |  |  |
| Proboscidea gen. et sp. indet. | + |  |  |  |  |
| *Phyllotillon huangheensis* (Qiu, 1998) | + | + |  |  |  |
| Indricotheriinae (Borissiak, 1923) | + | + |  |  |  |
| *Schizotherium ordosium* (Hu, 1959) |  |  |  |  |  |
| *Aprotodon lanzhouensis* (Qiu et Xie, 1997) | + | + | + |  |  |
| *Aprotodon* sp. |  |  |  |  |  |
| *Paraentelodon* sp. | + |  |  |  |  |
| Cervidae et sp. indet. |  | + |  |  |  |
| *Stephanocemas* sp. |  |  |  |  | + |
| Bovidae gen. et sp. indet. | + | + |  |  |  |

Supplementary Table S2**.** Paleomagnetic results from the Duitinggou section.

Stratigraphic level: 0 m presents top.

| Stratigraphiclevel（m） | Ts  (ºC) | Te  (ºC) | N | Dec  (º) | Inc  (º) | VGP lat (º) | MAD  (º) |
| --- | --- | --- | --- | --- | --- | --- | --- |
| 0.3 | 580 | 680 | 7 | 16.8 | 15.8 | 57.95 | 3.3 |
| 1.6 | 250 | 600 | 10 | 359.2 | 24.5 | 66.61 | 2.9 |
| 2 | 250 | 500 | 6 | 353.5 | 12.4 | 59.48 | 4.3 |
| 2.6 | 250 | 640 | 12 | 335.2 | 26.4 | 58.56 | 2.7 |
| 3 | 250 | 600 | 10 | 3.6 | 19.4 | 63.57 | 2.3 |
| 3.6 | 400 | 580 | 6 | 343.6 | 9.1 | 54.96 | 3.9 |
| 4.6 | 250 | 500 | 6 | 6.2 | 8.4 | 57.51 | 3.2 |
| 5.3 | 250 | 580 | 9 | 339.4 | 3.2 | 50.49 | 2.1 |
| 5.6 | 250 | 580 | 9 | 355.2 | 6.9 | 56.95 | 2.7 |
| 6 | 250 | 620 | 11 | 344.6 | 14.3 | 57.81 | 2.6 |
| 6.3 | 250 | 525 | 7 | 352.7 | 24.1 | 65.50 | 3.8 |
| 7 | 250 | 580 | 9 | 349.6 | 10.4 | 57.59 | 2.7 |
| 7.3 | 250 | 580 | 9 | 5.5 | 33.1 | 71.20 | 1.9 |
| 8 | 250 | 655 | 13 | 330.1 | 15.6 | 50.75 | 1.5 |
| 8.6 | 350 | 525 | 5 | 353.6 | 4.7 | 55.63 | 2.8 |
| 10 | 250 | 680 | 15 | 357 | 9 | 58.20 | 2.2 |
| 11 | 250 | 450 | 5 | 356.5 | 3.2 | 55.24 | 1.2 |
| 11.3 | 250 | 580 | 9 | 356.3 | 7.9 | 57.58 | 1.8 |
| 11.6 | 250 | 680 | 15 | 30.6 | 29.6 | 56.04 | 5.6 |
| 13 | 250 | 600 | 10 | 344.9 | 28 | 64.77 | 3 |
| 13.3 | 400 | 550 | 5 | 336 | 1.2 | 48.01 | 1.4 |
| 13.6 | 250 | 655 | 12 | 339.1 | 44.2 | 69.42 | 3 |
| 15.3 | 400 | 620 | 8 | 166.3 | -28.3 | -65.58 | 3.5 |
| 15.6 | 525 | 680 | 9 | 185.1 | -17 | -62.09 | 0.8 |
| 16 | 250 | 670 | 14 | 177.6 | -20.8 | -64.45 | 2 |
| 16.3 | 300 | 680 | 14 | 200.7 | -5.7 | -51.56 | 2.1 |
| 16.6 | 250 | 640 | 12 | 192.9 | -23.5 | -63.38 | 3.9 |
| 17 | 250 | 680 | 15 | 172 | -26 | -66.38 | 2.3 |
| 17.3 | 250 | 670 | 14 | 176.3 | -25.4 | -66.90 | 1.8 |
| 17.6 | 250 | 670 | 14 | 164.6 | -30.7 | -66.04 | 1.4 |
| 18 | 250 | 580 | 9 | 186.5 | -30.2 | -69.20 | 2.5 |
| 18.3 | 250 | 550 | 8 | 195.9 | -42.4 | -72.03 | 6.3 |
| 19 | 250 | 525 | 7 | 173.7 | -23.8 | -65.56 | 2.4 |
| 21 | 550 | 655 | 6 | 161.9 | -28.6 | -63.55 | 6.3 |
| 22.3 | 250 | 670 | 14 | 179.8 | -10.7 | -59.19 | 2.1 |
| 23 | 250 | 670 | 14 | 165.4 | -45.1 | -74.32 | 2.7 |
| 23.6 | 250 | 580 | 9 | 5.6 | 30.7 | 69.71 | 2.4 |
| 24.3 | 250 | 525 | 7 | 5.7 | 39.2 | 75.13 | 3.5 |
| 25 | 250 | 500 | 6 | 16.2 | 19.3 | 59.89 | 6.3 |
| 25.6 | 250 | 525 | 7 | 29.7 | 25.2 | 54.84 | 3.5 |
| 26.3 | 250 | 525 | 7 | 8.5 | 30.7 | 68.93 | 2.4 |
| 27.6 | 250 | 500 | 6 | 356.4 | 27.7 | 68.26 | 4.2 |
| 28.3 | 250 | 655 | 13 | 347.7 | 19.4 | 61.50 | 2 |
| 29.6 | 300 | 500 | 5 | 0.9 | 40.3 | 76.74 | 3.4 |
| 30.3 | 300 | 525 | 6 | 1.8 | 8.1 | 57.82 | 2.7 |
| 31 | 400 | 580 | 6 | 20.9 | 41.1 | 67.98 | 3.6 |
| 34.3 | 250 | 655 | 13 | 358.1 | 45.7 | 80.78 | 2.8 |
| 35 | 250 | 525 | 7 | 56.2 | 40.8 | 40.26 | 3.9 |
| 35.6 | 250 | 620 | 11 | 21 | 60 | 72.97 | 2.2 |
| 36.3 | 250 | 600 | 10 | 350.3 | 23.1 | 64.29 | 2.9 |
| 37 | 250 | 640 | 12 | 23.3 | 43.1 | 67.16 | 5.1 |
| 37.6 | 250 | 655 | 13 | 3 | 46.5 | 81.20 | 3 |
| 38.3 | 250 | 600 | 10 | 15.1 | 18.6 | 60.02 | 3 |
| 39 | 250 | 600 | 10 | 21.1 | 49.2 | 71.35 | 3.4 |
| 39.6 | 250 | 655 | 13 | 4.3 | 48.3 | 82.20 | 1.6 |
| 40.3 | 250 | 655 | 13 | 348.9 | 36.7 | 71.48 | 2.5 |
| 41 | 250 | 655 | 13 | 10.5 | 37.6 | 72.29 | 3.7 |
| 41.6 | 250 | 655 | 13 | 5.7 | 51.6 | 83.84 | 2.6 |
| 42.3 | 250 | 580 | 9 | 8.1 | 39.9 | 74.76 | 3.5 |
| 43.6 | 250 | 655 | 13 | 10.7 | 50.2 | 79.67 | 3.4 |
| 44.3 | 300 | 640 | 11 | 5 | 36.7 | 73.63 | 2.8 |
| 45 | 250 | 655 | 13 | 15.5 | 43.9 | 73.09 | 2.1 |
| 45.6 | 250 | 580 | 9 | 12.4 | 34.5 | 69.59 | 5.1 |
| 46.3 | 250 | 655 | 13 | 349.8 | 44.4 | 76.65 | 2.6 |
| 47 | 250 | 525 | 7 | 1.6 | 46.8 | 81.71 | 2.7 |
| 47.6 | 350 | 500 | 4 | 349.6 | 23.3 | 64.18 | 1.6 |
| 48.3 | 250 | 580 | 9 | 1.5 | 35 | 73.03 | 3.8 |
| 49 | 250 | 680 | 15 | 11.7 | 36.3 | 70.96 | 5 |
| 49.6 | 300 | 580 | 8 | 20.8 | 45.7 | 70.16 | 2.7 |
| 50.3 | 250 | 580 | 9 | 11.5 | 13.6 | 58.86 | 5.8 |
| 51 | 250 | 500 | 6 | 13 | 32.8 | 68.35 | 1.6 |
| 51.6 | 250 | 655 | 13 | 338.8 | 24.2 | 59.71 | 3.5 |
| 54.3 | 400 | 580 | 6 | 45.2 | 30.1 | 45.28 | 4.8 |
| 55 | 300 | 525 | 6 | 20.7 | 16.8 | 56.56 | 3 |
| 55.6 | 250 | 655 | 12 | 12.6 | 21.7 | 62.57 | 3 |
| 56.3 | 250 | 500 | 6 | 28.7 | 20.4 | 53.49 | 2.2 |
| 57.6 | 250 | 500 | 6 | 359.4 | 9.9 | 58.77 | 1.7 |
| 58.3 | 250 | 500 | 6 | 358.1 | 4.6 | 56.05 | 1.5 |
| 59 | 350 | 620 | 9 | 350 | 33.3 | 69.93 | 3.2 |
| 59.6 | 350 | 580 | 7 | 358.7 | 5.8 | 56.67 | 2.2 |
| 60.3 | 400 | 600 | 6 | 22.1 | 14.8 | 54.93 | 3.6 |
| 61.6 | 250 | 680 | 15 | 229.7 | -22.8 | -39.21 | 1.7 |
| 62.3 | 250 | 500 | 6 | 194.4 | -45.3 | -74.56 | 1.7 |
| 63 | 450 | 600 | 6 | 177.4 | -34.2 | -72.41 | 4.6 |
| 65 | 250 | 670 | 14 | 175.7 | -23.3 | -65.63 | 3.2 |
| 65.6 | 500 | 620 | 5 | 188 | -49.9 | -81.35 | 4.6 |
| 67 | 600 | 670 | 5 | 168.5 | -28 | -66.33 | 4.3 |
| 67.6 | 525 | 600 | 4 | 181.5 | -43.7 | -79.25 | 3.2 |
| 68.3 | 250 | 500 | 6 | 199.9 | -29.1 | -62.78 | 4.8 |
| 69 | 250 | 525 | 7 | 174.2 | -29.4 | -68.88 | 2.8 |
| 69.6 | 250 | 580 | 9 | 183.2 | -38.2 | -75.01 | 1.6 |
| 70.3 | 250 | 580 | 9 | 205.9 | -33.2 | -60.87 | 2.4 |
| 71 | 250 | 670 | 14 | 178.4 | -11.4 | -59.51 | 1.9 |
| 71.42 | 250 | 670 | 14 | 216.1 | -25.5 | -50.46 | 2.2 |
| 71.91 | 550 | 680 | 8 | 190.8 | -20.7 | -62.68 | 1.7 |
| 72.82 | 250 | 680 | 15 | 162.6 | -25.7 | -62.47 | 2.1 |
| 73.31 | 250 | 680 | 15 | 191 | -23.3 | -63.98 | 2.4 |
| 73.8 | 250 | 500 | 6 | 180.5 | -44.3 | -79.79 | 1.7 |
| 74.22 | 350 | 580 | 7 | 168 | -55.5 | -80.31 | 4 |
| 74.71 | 600 | 680 | 6 | 187 | -63.9 | -79.25 | 2.1 |
| 75.2 | 250 | 670 | 14 | 188.5 | -32.5 | -69.99 | 1.6 |
| 75.62 | 525 | 670 | 8 | 193.3 | -36.8 | -70.46 | 2.3 |
| 76.11 | 250 | 670 | 14 | 191.2 | -33.4 | -69.50 | 1.9 |
| 76.6 | 250 | 670 | 14 | 201.7 | -49.4 | -70.95 | 2.5 |
| 77.02 | 250 | 500 | 6 | 183.6 | -34 | -72.14 | 1.6 |
| 77.51 | 250 | 670 | 14 | 166.6 | -35.6 | -69.73 | 3.3 |
| 78 | 250 | 670 | 14 | 199.1 | -35.7 | -66.53 | 2.5 |
| 78.42 | 250 | 655 | 13 | 329 | 21 | 52.24 | 1.9 |
| 78.91 | 250 | 670 | 14 | 20.5 | 8.9 | 53.09 | 2.3 |
| 79.4 | 525 | 680 | 9 | 358.7 | 21.1 | 64.68 | 2.4 |
| 79.82 | 250 | 670 | 14 | 350.3 | 12.8 | 58.97 | 2.3 |
| 80.31 | 250 | 670 | 14 | 335.6 | 10.8 | 51.96 | 1.6 |
| 80.8 | 250 | 670 | 14 | 359 | 42.4 | 78.30 | 1.6 |
| 81.22 | 250 | 680 | 15 | 333.6 | 20.7 | 55.07 | 2.2 |
| 81.71 | 250 | 580 | 9 | 6.5 | 29 | 68.49 | 2.2 |
| 82.2 | 250 | 680 | 15 | 35.3 | 16.2 | 47.44 | 1.1 |
| 82.62 | 250 | 600 | 10 | 8.4 | 8.4 | 57.09 | 1.5 |
| 83.11 | 250 | 500 | 6 | 4.9 | 19.3 | 63.35 | 2.8 |
| 83.6 | 250 | 500 | 6 | 8.9 | 42.7 | 76.24 | 3 |
| 85 | 400 | 670 | 11 | 12.9 | 49 | 77.50 | 2 |
| 85.42 | 250 | 655 | 13 | 329.7 | 28.7 | 55.88 | 3.6 |
| 85.91 | 300 | 525 | 6 | 18.9 | 19.9 | 58.91 | 5.1 |
| 86.4 | 600 | 670 | 5 | 24.6 | 11.1 | 51.98 | 3 |
| 87.1 | 300 | 580 | 8 | 314.5 | 18.4 | 40.90 | 6.3 |
| 87.52 | 300 | 670 | 13 | 355.5 | 57.6 | 85.89 | 3 |
| 87.8 | 250 | 600 | 10 | 0.5 | 11 | 59.34 | 2 |
| 88.22 | 250 | 670 | 14 | 333 | 37.3 | 61.90 | 2.1 |
| 88.5 | 250 | 640 | 12 | 4.7 | 17.9 | 62.63 | 2.4 |
| 88.92 | 250 | 670 | 14 | 350.1 | 41.6 | 75.05 | 1.8 |
| 89.2 | 250 | 500 | 6 | 355.8 | 12.4 | 59.82 | 3 |
| 89.62 | 250 | 680 | 15 | 18.4 | 31.8 | 64.98 | 2 |
| 89.9 | 350 | 620 | 9 | 32.1 | 51.9 | 63.37 | 4.4 |
| 90.32 | 300 | 680 | 14 | 10.1 | 47.3 | 78.48 | 3.1 |
| 90.6 | 250 | 620 | 11 | 6.1 | 46.1 | 79.83 | 3 |
| 91.02 | 300 | 680 | 14 | 0.5 | 49.7 | 84.30 | 1.8 |
| 91.3 | 250 | 620 | 11 | 0.4 | 28.1 | 68.73 | 4.7 |
| 91.72 | 250 | 680 | 15 | 350.2 | 25.9 | 65.78 | 2 |
| 92 | 250 | 680 | 15 | 336.3 | 28.7 | 60.29 | 3 |
| 92.42 | 525 | 655 | 7 | 26.2 | 47.1 | 66.55 | 4.4 |
| 92.7 | 250 | 680 | 15 | 24.7 | 37.3 | 63.55 | 2.3 |
| 93.12 | 300 | 680 | 14 | 0.8 | 39.7 | 76.32 | 4.5 |
| 93.4 | 350 | 670 | 12 | 350.7 | 45.8 | 78.03 | 4.4 |
| 93.82 | 350 | 680 | 13 | 348.5 | 47.8 | 77.86 | 2.2 |
| 94.1 | 350 | 600 | 8 | 6.2 | 32.8 | 70.85 | 2.7 |
| 94.52 | 250 | 580 | 10 | 9.4 | 45.4 | 77.72 | 2.5 |
| 97.6 | 300 | 580 | 8 | 8.3 | 57.6 | 83.09 | 1.7 |
| 98.02 | 300 | 620 | 10 | 217.2 | -28.7 | -50.88 | 1.8 |
| 98.3 | 350 | 655 | 11 | 205 | -32.9 | -61.35 | 2.7 |
| 98.72 | 250 | 655 | 13 | 208 | -29.6 | -57.86 | 3.1 |
| 99 | 250 | 670 | 14 | 240.6 | -45.7 | -38.45 | 1.4 |
| 99.42 | 250 | 670 | 14 | 219.3 | -39.7 | -53.43 | 2.5 |
| 99.7 | 250 | 525 | 7 | 214.9 | -47.2 | -59.65 | 3.1 |
| 100.12 | 250 | 670 | 14 | 239.4 | -49.7 | -40.83 | 1.4 |
| 100.82 | 600 | 670 | 5 | 200.7 | -47.1 | -70.83 | 1.9 |
| 101.1 | 350 | 580 | 7 | 208.6 | -37.4 | -60.77 | 3 |
| 101.52 | 400 | 670 | 11 | 207.2 | -4.8 | -47.88 | 3.4 |
| 101.8 | 300 | 655 | 12 | 194.5 | -6.6 | -54.48 | 4.2 |
| 102.22 | 300 | 680 | 14 | 177.2 | -21.5 | -64.80 | 3 |
| 102.5 | 300 | 655 | 12 | 172.7 | -37.1 | -73.24 | 2 |
| 102.92 | 450 | 670 | 10 | 156 | -29.6 | -60.51 | 2.7 |
| 103.2 | 250 | 670 | 14 | 153.1 | -39.8 | -63.05 | 1.7 |
| 103.62 | 400 | 680 | 12 | 175.1 | -61.6 | -82.44 | 2.1 |
| 103.9 | 300 | 600 | 9 | 206.8 | -63 | -68.09 | 3.1 |
| 104.6 | 250 | 670 | 14 | 230.8 | -6 | -32.7 | 1.6 |
| 104.81 | 250 | 640 | 12 | 234.7 | -54.5 | -46.13 | 3.5 |
| 105.3 | 250 | 670 | 14 | 189.5 | -34.7 | -70.96 | 1.6 |
| 105.51 | 250 | 600 | 10 | 205.8 | -30.4 | -59.69 | 2.4 |
| 106 | 250 | 670 | 14 | 227.2 | -37.6 | -46.37 | 1.7 |
| 106.21 | 250 | 680 | 15 | 207 | -49.1 | -66.63 | 1.3 |
| 106.7 | 250 | 500 | 6 | 230.1 | -46.6 | -47.19 | 2.1 |
| 106.91 | 250 | 670 | 14 | 172.7 | -20 | -63.27 | 2.2 |
| 107.4 | 250 | 670 | 14 | 178.5 | -18.9 | -63.47 | 1.6 |
| 107.61 | 400 | 670 | 11 | 170.4 | -10.6 | -57.90 | 2 |
| 108.1 | 250 | 670 | 14 | 185.3 | -40.7 | -76.27 | 1.6 |
| 108.31 | 250 | 670 | 14 | 158.6 | -21.8 | -58.48 | 2.1 |
| 108.8 | 250 | 670 | 14 | 173.5 | -40.3 | -75.62 | 2.3 |
| 109.01 | 250 | 620 | 11 | 199 | -47.8 | -72.42 | 3.3 |
| 109.71 | 250 | 670 | 14 | 221.7 | -43.6 | -52.93 | 1.2 |
| 110.2 | 250 | 580 | 9 | 218.5 | -44.2 | -55.71 | 2.3 |
| 110.41 | 350 | 620 | 9 | 194.1 | -53.8 | -78.35 | 5.5 |
| 111.11 | 250 | 670 | 14 | 313.7 | 23.3 | 42.01 | 1.9 |
| 111.81 | 250 | 580 | 9 | 1.2 | 24.1 | 66.37 | 2.6 |
| 112.3 | 250 | 680 | 15 | 333.5 | 22.5 | 55.78 | 1.6 |
| 112.51 | 250 | 670 | 14 | 317.5 | 17.2 | 42.71 | 3.5 |
| 113 | 250 | 680 | 15 | 46.3 | 47 | 50.39 | 1.4 |
| 113.21 | 300 | 580 | 8 | 348.8 | 27.9 | 66.39 | 2.7 |
| 113.7 | 250 | 580 | 9 | 342 | 40.1 | 69.47 | 4.1 |
| 113.91 | 250 | 640 | 12 | 18.6 | 46.5 | 72.16 | 1.9 |
| 114.4 | 250 | 670 | 14 | 39.2 | 26 | 48.37 | 3.1 |
| 114.61 | 250 | 640 | 12 | 2.7 | 33.1 | 71.69 | 4.2 |
| 115.1 | 250 | 680 | 15 | 18 | 21.5 | 60.12 | 3.5 |
| 115.31 | 250 | 580 | 9 | 3.8 | 18.9 | 63.28 | 4.7 |
| 115.8 | 200 | 500 | 7 | 2.1 | 25.3 | 67.01 | 5 |
| 116.01 | 350 | 580 | 6 | 4.8 | 49.7 | 83.04 | 3.8 |
| 116.71 | 350 | 670 | 12 | 3.3 | 23.7 | 65.98 | 3.3 |
| 116.92 | 250 | 680 | 15 | 17.4 | 25 | 62.12 | 4 |
| 117.92 | 250 | 680 | 15 | 3.7 | 26.2 | 67.37 | 1.4 |
| 121.92 | 250 | 655 | 13 | 340 | 34.7 | 65.47 | 6.7 |
| 122.62 | 450 | 655 | 9 | 11.8 | 30.2 | 67.43 | 6.3 |
| 122.92 | 250 | 580 | 9 | 348.3 | 5.1 | 54.65 | 3 |
| 124.92 | 580 | 670 | 6 | 134.7 | -35.4 | -47.10 | 4.6 |
| 125.62 | 300 | 670 | 13 | 170.4 | -44.4 | -76.98 | 1.5 |
| 125.92 | 250 | 580 | 9 | 179.3 | -65.2 | -78.94 | 1.3 |
| 126.32 | 250 | 620 | 11 | 146.6 | -50.1 | -61.80 | 2.8 |
| 130.92 | 400 | 670 | 11 | 226.1 | -45.5 | -50.04 | 2.2 |
| 131.92 | 250 | 670 | 14 | 213.4 | -43.9 | -59.67 | 1.9 |
| 132.32 | 250 | 640 | 12 | 210.9 | -51.8 | -64.31 | 1.7 |
| 132.92 | 350 | 600 | 8 | 206 | -37.3 | -62.62 | 3.1 |
| 133.32 | 300 | 580 | 8 | 200.9 | -38.3 | -66.64 | 3.8 |
| 133.92 | 350 | 580 | 7 | 205.3 | -44.7 | -66.32 | 5.7 |
| 134.92 | 500 | 680 | 10 | 183 | -38.9 | -75.52 | 3.9 |
| 143.92 | 525 | 670 | 8 | 215.7 | -43.7 | -57.77 | 3.1 |
| 144.92 | 250 | 640 | 12 | 206.8 | -28.2 | -58.06 | 2.3 |
| 145.92 | 250 | 525 | 7 | 220.2 | -37.1 | -51.76 | 4 |
| 146.32 | 250 | 580 | 9 | 217.4 | -38.2 | -54.37 | 1.7 |
| 147.32 | 250 | 450 | 5 | 333.7 | 33.5 | 60.73 | 2 |
| 148.32 | 300 | 500 | 5 | 234.2 | -60.6 | -48.17 | 2.3 |
| 148.62 | 250 | 500 | 6 | 200.3 | -46.2 | -70.76 | 4.7 |
| 150.32 | 400 | 670 | 11 | 179.8 | -34.4 | -72.69 | 2 |
| 150.62 | 250 | 580 | 9 | 216.7 | -32.1 | -52.57 | 1.1 |
| 151.32 | 300 | 640 | 11 | 213.7 | -27.8 | -53.10 | 2.4 |
| 151.62 | 250 | 580 | 9 | 193.2 | -51.1 | -78.21 | 3.1 |
| 152.32 | 250 | 680 | 15 | 217.1 | -34.3 | -53.11 | 1.2 |
| 152.62 | 450 | 620 | 7 | 219.3 | -33.4 | -51.07 | 1.5 |
| 153.32 | 250 | 580 | 9 | 20.5 | 50.6 | 72.31 | 3.1 |
| 153.62 | 250 | 670 | 14 | 3.9 | 45.3 | 80.03 | 3 |
| 154.32 | 250 | 580 | 9 | 12.5 | 42.6 | 74.26 | 2.4 |
| 154.62 | 300 | 450 | 4 | 2.9 | 39.1 | 75.68 | 4 |
| 155.32 | 250 | 680 | 15 | 195.2 | -46 | -74.38 | 0.8 |
| 155.62 | 250 | 670 | 14 | 190.2 | -51.6 | -80.69 | 1.6 |
| 156.62 | 250 | 680 | 15 | 1.3 | 27.7 | 68.47 | 2.6 |
| 157.32 | 250 | 680 | 15 | 343.2 | 30.6 | 65.26 | 1.1 |
| 157.62 | 250 | 670 | 14 | 332.1 | 13.2 | 50.99 | 2 |
| 158.32 | 250 | 680 | 15 | 14.3 | 46.7 | 75.36 | 2.2 |
| 158.62 | 250 | 580 | 9 | 35.1 | 38.2 | 56.16 | 4.8 |
| 160.32 | 250 | 655 | 13 | 201.2 | -54.5 | -72.76 | 2.4 |
| 160.62 | 250 | 620 | 11 | 217.1 | -45.1 | -57.15 | 5.8 |
| 161.62 | 250 | 670 | 14 | 355 | 20.8 | 64.14 | 1.7 |
| 162.32 | 250 | 655 | 13 | 322.8 | 31.8 | 52.07 | 2.3 |
| 162.62 | 350 | 670 | 12 | 326.5 | 33.2 | 55.40 | 2.5 |
| 163.62 | 250 | 580 | 9 | 348.8 | 23 | 63.75 | 3.5 |
| 164.62 | 250 | 580 | 9 | 2 | 53.3 | 87.13 | 5.5 |
| 165.62 | 250 | 580 | 9 | 16.2 | 48.6 | 74.88 | 5 |
| 166.62 | 300 | 620 | 10 | 357.4 | 46.8 | 81.53 | 1.9 |
| 167.62 | 250 | 640 | 12 | 28.9 | 40.1 | 61.67 | 2.7 |
| 169.62 | 250 | 655 | 13 | 166.1 | -45.2 | -74.85 | 2.3 |
| 170.62 | 450 | 670 | 10 | 180.3 | -23.5 | -66.05 | 1 |
| 171.62 | 450 | 655 | 9 | 175.4 | -32.3 | -70.89 | 3.3 |
| 172.62 | 250 | 680 | 15 | 166.5 | -1.8 | -52.54 | 1.6 |
| 173.62 | 300 | 580 | 8 | 185.7 | -29.9 | -69.20 | 2.2 |
| 174.62 | 300 | 580 | 8 | 173.3 | -36.2 | -72.85 | 2.7 |
| 179.62 | 400 | 580 | 6 | 194.4 | -47.1 | -75.49 | 1.5 |
| 181.62 | 250 | 600 | 10 | 318.1 | 22.4 | 45.03 | 6.7 |
| 182.62 | 300 | 670 | 13 | 352 | 46.6 | 79.26 | 1.3 |
| 183.62 | 250 | 655 | 13 | 5.9 | 58.5 | 84.45 | 2.6 |
| 184.62 | 350 | 525 | 5 | 215.9 | -50 | -59.75 | 2.6 |
| 186.62 | 250 | 640 | 12 | 202.7 | -19.6 | -56.76 | 1.9 |
| 187.62 | 350 | 600 | 8 | 183 | -58.2 | -86.42 | 2.3 |
| 189.62 | 500 | 600 | 5 | 55.3 | 35.8 | 39.23 | 5 |
| 190.62 | 250 | 670 | 14 | 1.2 | 45.8 | 80.94 | 1.1 |
| 191.92 | 450 | 600 | 6 | 3 | 10.6 | 59.01 | 2.2 |
| 192.92 | 525 | 670 | 8 | 6.3 | 28.5 | 68.24 | 3.1 |
| 196.62 | 450 | 580 | 5 | 218.7 | -18.4 | -45.90 | 3.9 |
| 203.92 | 300 | 655 | 12 | 197.2 | -15.2 | -57.49 | 2.6 |
| 204.92 | 400 | 580 | 6 | 212.1 | -41.4 | -59.74 | 2.9 |
| 205.92 | 620 | 670 | 4 | 186.1 | -33.1 | -71.06 | 5.3 |
| 206.92 | 500 | 600 | 5 | 236.4 | -41.8 | -40.45 | 5.8 |
| 211.92 | 500 | 670 | 9 | 203.1 | -45 | -68.13 | 1.9 |
| 212.62 | 500 | 670 | 9 | 193.7 | -29.9 | -66.44 | 2 |

Notes:

Ts and Te: lower and upper temperature steps of the characteristic remanent magnetization (ChRM) trajectory;

N: number of consecutive points/steps used to determine the ChRM directions;

Dec and Inc: declination and inclination of the ChRM direction;

VGP lat.: Virtual geomagnetic pole latitude derived from the ChRM direction;

MAD: Maximum angular deviation of ChRM direction in degrees.

Supplementary Table S3**.** Parameter values of IRM Coercivity components of the modelled samples.

| Depth | Component | SIRM  (absolute) | Log(B_1/2_) | B_1/2_(mT) | Contribution of IRM (%) | DP |
| --- | --- | --- | --- | --- | --- | --- |
| 48.3 m | 1 | 2.80 | 1.65 | 45.6 | 55 | 0.42 |
|  | 2 | 2.27 | 3.43 | 2672.7 | 45 | 1.28 |
| 71.6 m | 1 | 0.87 | 1.49 | 31.1 | 32 | 0.26 |
|  | 2 | 1.83 | 2.63 | 425.0 | 68 | 0.88 |
| 155.6 m | 1 | 1.10 | 1.50 | 31.7 | 37 | 0.28 |
|  | 2 | 1.90 | 2.48 | 302.4 | 63 | 0.78 |

Notes: SIRM, saturation IRM; B_1/2_, the field at which half of the SIRM is reached; DP, the dispersion of the distribution.

**References for Supplementary Material:**

1. Opdyke, N. D., Flynn, L. J., Lindsay, E. H., & Qiu, Z. X. The magnetic stratigraphy of the Yehucheng and Xianshuihe Formations of Oligocene- Miocene age near Lanzhou city, China in *Proceedings, Paleontology Working Meeting* 51 (Lanzhou, China, 1998)

2. Flynn, L. J. *et al.* Recent advances in the small mammal biostratigraphy and magnetostratigraphy of Lanzhou Basin. *Chin. Sci. Bull.* **44**, 105–118 (1999).

3. Qiu, Z. X. *et al.* Neogene Land Mammal Stages/Ages of China in *Fossil Mammals of Asia: Neogene Biostratigraphy and Chronology* (eds Wang, X. M., Flynn, L. J. & Fortelius, M.) 29–90 (New York, Columbia University Press, 2013)

4. Hilgen, F., Lourens, L. & Van Dam, J. The Neogene Period in *The Geologic Time Scale* (eds Gradstein, F. M., Ogg, J. G., Schmitz, M. D., & Ogg, G. M.) 923–978 (Amsterdam, Elsevier, 2012)

5. Vandenberghe, N., Hilgen, F. & Speijer, R. The Paleogene period in *The Geologic Time Scale* (eds Gradstein, F. M., Ogg, J. G., Schmitz, M. D. & Ogg, G. M.) 855–921 (Amsterdam, the Netherlands, Elsevier, 2012)

6. Ao, H., Dekkers, M. J., Deng, C. L. & Zhu, R. X. Palaeclimatic significance of the Xiantai fluvio-lacustrine sequence in the Nihewan Basin (North China), based on rock magnetic properties and clay mineralogy. *Geophys. J. Int.* **177**, 913–924 (2009).

7. Ao, H., Deng, C. L., Dekkers, M. J. & Liu, Q. S. Magnetic mineral dissolution in Pleistocene fluvio-lacustrine sediments, Nihewan Basin (North China). *Earth Planet. Sci. Lett.* **292**, 191–200 (2010).

8. McFadden, P. L. & McElhinny, M. W. Classification of the reversal test in palaeomagnetism. *Geophys. J. Int.* **103**, 725–729 (1990).

9. Xiao, G. Q. et al. Evidence for northeastern Tibetan Plateau uplift between 25 and 20 Ma in the sedimentary archive of the Xining Basin, Northwestern China. *Earth Planet. Sci. Lett.* **317–318**, 185–195 (2012).

10. Dupont-Nivet, G., Dai, S., Fang, X. M., Krijgsman, W., Erens, V., Reitsma, M., Langereis, C. Timing and distribution of tectonic rotations in the northeastern Tibetan Plateau. *Geol. Soc. Am.* *Spec. Pap.* **444**, 73–87 (2008).

11. Dupont-Nivet, G. *et al.* Paleogene clockwise tectonic rotation of the Xining–Lanzhou region, northeastern Tibetan Plateau. *J. Geophys. Res.* **109**, B04401 (2004).

12. Yan, M. D., Van der Voo, R., Tauxe, L., Fang, X. M. & Parés, J. M. Shallow bias in Neogene palaeomagnetic directions from the Guide Basin, NE Tibet, caused by inclination error. *Geophys. J. Int.* **163**, 944–948 (2005).

13. Arason, P. & Levi, S. Models of inclination shallowing during sediment compaction. J. *Geophys. Res.* **95**, 4481–4499 (1990).

14. Gilder, S., Chen, Y. & Sen, S. Oligo-Miocene magnetostratigraphy and rock magnetism of the Xishuigou section, Subei (Gansu Province, western China) and implications for shallow inclinations in central Asia. *J. Geophys. Res.* **106**, 30505–30521 (2001).

15. Tauxe, L. & Kent, D. V. A simplified statistical model for the geomagnetic field and the detection of shallow bias in paleomagnetic inclinations: was the ancient magnetic field dipolar? in *Timescales of the Paleomagnetic field* (eds Channell, J. E. T. Kent, D. V. Lowrie, W. & Meert, J. G.) 101–115 (American Geophysical Union, 2004).

16. Qiu, Z. X. *et al.* Recent advances in study of the Xianshuihe Formation in Lanzhou Basin in *Evidence for Evolution–Essays in Honor of Prof. Chungchien Young on the Hundredth Anniversary of His Birth* (ed Tong, Y. S.) 177–192 (Beijing, China Ocean Press, 1997). (in Chinese)

17. Xie, G. P. The Tertiary and local mammalian faunas in Lanzhou Basin, Gansu. *J. stratigr.* **28**, 67–80 (2004). (in Chinese with English abstract)

18. Li, Z. C., Li, Y. X., Zhang, Y. X., Li, W. H. & Xie, K. Nanpoping fauna of the Lanzhou Basin and its environmental significance: *Sci. China Earth Sci.* **59**, 1258–1266 (2016).
